# RNA-Mediated Regulation of Glycolysis in Embryonic Stem Cell Pluripotency and Differentiation

**DOI:** 10.1007/s40778-024-00236-9

**Published:** 2024-05-09

**Authors:** Wei Fan, Xiaoling Li

**Affiliations:** 1 Signal Transduction Laboratory, National Institute of Environmental Health Sciences, North Carolina, Research Triangle Park, Triangle Park, USA

**Keywords:** Embryonic stem cells, glycolysis, Metabolic shift, Pluripotency, Differentiation, RNA

## Abstract

**Purpose of Review:**

The maintenance and differentiation of embryonic stem cells are strictly coordinated with their metabolic status. As a core part of cellular metabolism, glycolysis provides energy and biomolecules important for stem cell proliferation and functions. Particularly, the differentiation of embryonic stem cells is associated with metabolic shifts between glycolysis and oxidative phosphorylation. However, how these metabolic swifts are regulated is still not completely known. This review aims to highlight recent advances in regulation of glycolysis at different stages of embryonic stem cells.

**Recent Findings:**

Through modulating glycolysis, multiple types of RNA molecules and RNA-binding proteins are critically involved in regulation of the self-renewal, pluripotency, and differentiation of embryonic stem cells.

**Summary:**

RNA-mediated glycolytic regulation in embryonic stem cells is important for their maintenance and transitions between different stages of differentiation. Better understanding of these riboregulatory mechanisms will have potential for future research and therapeutic innovation.

## Introduction

The Embryonic stem cells (ESCs) are cell lines derived from the inner cell mass of the mammalian blastocyst. The important functional features of ESCs include high capacity of self-renewal to infinitely self-replicate and pluripotency with broad differentiation plasticity into three different germ layers of endoderm, mesoderm, or ectoderm [1]. The stemness maintenance and differentiation of ESCs are regulated by multiple precisely orchestrated circuitries comprising molecular machineries like transcription, translation and epigenetics [2]. Mouse(m) and human(h) ESCs utilize distinct signaling pathways to regulate maintenance of pluripotency, self-renew and normal differentiation process. For instance, mESCs maintain their stemness by the leukemia inhibitory factor (LIF)/Signal transducer and activator of transcription 3 (STAT3) signal pathway, whereas the stemness maintenance and early lineage specification of primed hESCs are dominantly regulated by both FGF2 and Acticin/Nodal signaling pathways [3–6]. Therefore, supplement or removal of LIF and inhibitors for GSK3β and Mek1/2 (2i) (for mESCs) or FGF2 (for hESCs) are commonly used for maintenance or differentiation of ESCs.

Both glycolysis and oxidative phosphorylation (OXPHOS) are fundamental cellular metabolic process. By consuming one glucose molecule, OXPHOS can generate 38 Adenosine Triphosphates (ATP) whereas glycolysis generates 2 ATPs. Beside generating direct energy ATP, glycolysis and OXPHOS also provide intermediate metabolites to synthesize cell basic building components, such as amino acids, nucleotides and lipids, to support cell proliferation, especially in highly self-renewing stem and cancer cells [7, 8]. Most importantly, these cellular metabolites are also the substrate/co-factors of epigenetic modifications in ESCs and critically involved in embryonic de novo genome activation [9].

Both mESCs and hESCs are predominantly rely on aerobic glycolysis, which provides direct energy source for activities of living organism and is crucial for regulation of stem cell self-renewal and differentiation. The glycolytic flux decreases when mESCs commit to embryonic germ layers, then OXPHOS becomes a more pivotal cellular energy source [1]. In hESCs, however, differentiation of different germ layers is associated with distinct metabolic features [10–12]. Specifically, glycolysis is decreased and OXPHOS is elevated for differentiation of mesoderm and endoderm, but a high glycolytic flux is maintained during ectoderm differentiation [13, 14]. Conversely, a reversed alteration of glycolytic metabolism occurs during the reprogramming of somatic cells to inducible pluripotent stem cells (iPSCs) [14]. For example, glycolysis is increased during the reprogramming of fibroblasts to iPSCs [15, 16]. Overall, stemness demands vibrant glycolysis whereas the lineage differentiation is associated with the shifting toward OXPHOS [14, 15].

Metabolic remodeling also occurs during the transitions between the naïve state (nESC) and primed states of ESCs. These two states are classifications of the pluripotency levels of ESCs, corresponding to the cellular states of the preimplantation mouse blastocyst inner cell mass and post-implantation epiblast stem cells (EpiSCs) in vivo, respectively [17]. ESCs cultured with 2i/LIF medium (containing LIF, GSK3β and Mek 1/2 inhibitors) maintains nESC states homogeneously. In contrast, ESCs cultured with serum containing medium are heterogeneous cells, comprising nESCs, EpiSCs and intermediate state epiblast-like cells (EpiLCs) [18]. Interestingly, maintenance of nESCs is dependent on a metabolic state combining glycolysis and OXPHOS (bivalent metabolism), and the transition from nESCs to EpiSCs is accompanied with shifting from this bivalent metabolism to exclusive glycolysis (glycolytic metabolism) [19]. This metabolic switch from a bivalent metabolism in naive state to predominantly glycolysis in primed state is a continuum feature of ESC pluripotent development [20]. It is worth noting that although nESCs are on a bivalent metabolic status with a greater reliance on OXPHOS [19], both the mRNA and protein levels of glycolytic enzymes are significantly higher in mESCs cultured in 2i medium compared to those cultured in serum [18, 21] and naïve hESCs have higher glycolysis and oxygen consumption compared to their primed counterparts [14], indicating that nESCs possess a higher overall metabolic flux (including glycolytic flux) than primed ESCs.

Collectively, glycolytic metabolism is a critical feature of pluripotent ESCs and may be functionally important in maintaining undifferentiated ESCs [22]. In recent years, multiple novel regulators/mediators that fine-tune the metabolic shift between stemness and differentiation states of ESCs have been discovered, including RNA and RNA-binding proteins (RBPs) [23]. Multiple types of RNA molecules, regardless of coding/non-coding or long/microRNA molecules, have emerged as important regulators or mediators in different signaling pathways affecting glycolytic metabolism and differentiation of ESCs [6, 24, 25••]. Through binding and regulation of RNAs, RBPs have been shown to facilitate metabolic shifting in cells undergoing differentiation process, thereby are critically involved in the regulation of pluripotency in stem cells [2]. Particularly, many glycolytic enzymes have been identified as RBPs in stem cells [25••], and some RBPs are important in mediating the expression of glycolytic enzymes in stem cells [26•]. This review article summarizes the latest knowledges on RNA-mediated regulation (riboregulation) of glycolysis in the self-renewal, pluripotency and differentiation of ESCs.

## Long Noncoding RNAs Interact with Core Pluripotency Factors to Regulate Glycolysis in ESCs

Core pluripotency factors, including SOX2, OCT4, NANOG, and c-MYC, have been shown to promote glycolysis in PSCs by directly regulating transcription of glycolytic enzymes and/or glucose transporters [14, 27–30]. Recently studies demonstrated that these core pluripotency factors also regulate glycolysis indirectly through long non-protein-coding RNA transcripts longer than 200 nucleotides (lncRNAs). Despite having no capability of protein synthesis, lncRNAs are able to regulate pivotal pluripotency factors, modulate chromatin modifications, or counteract microRNA(miRNA) in stem cells. They are functionally important in embryogenesis and ESCs [22, 31, 32].

**Fig. 1:**
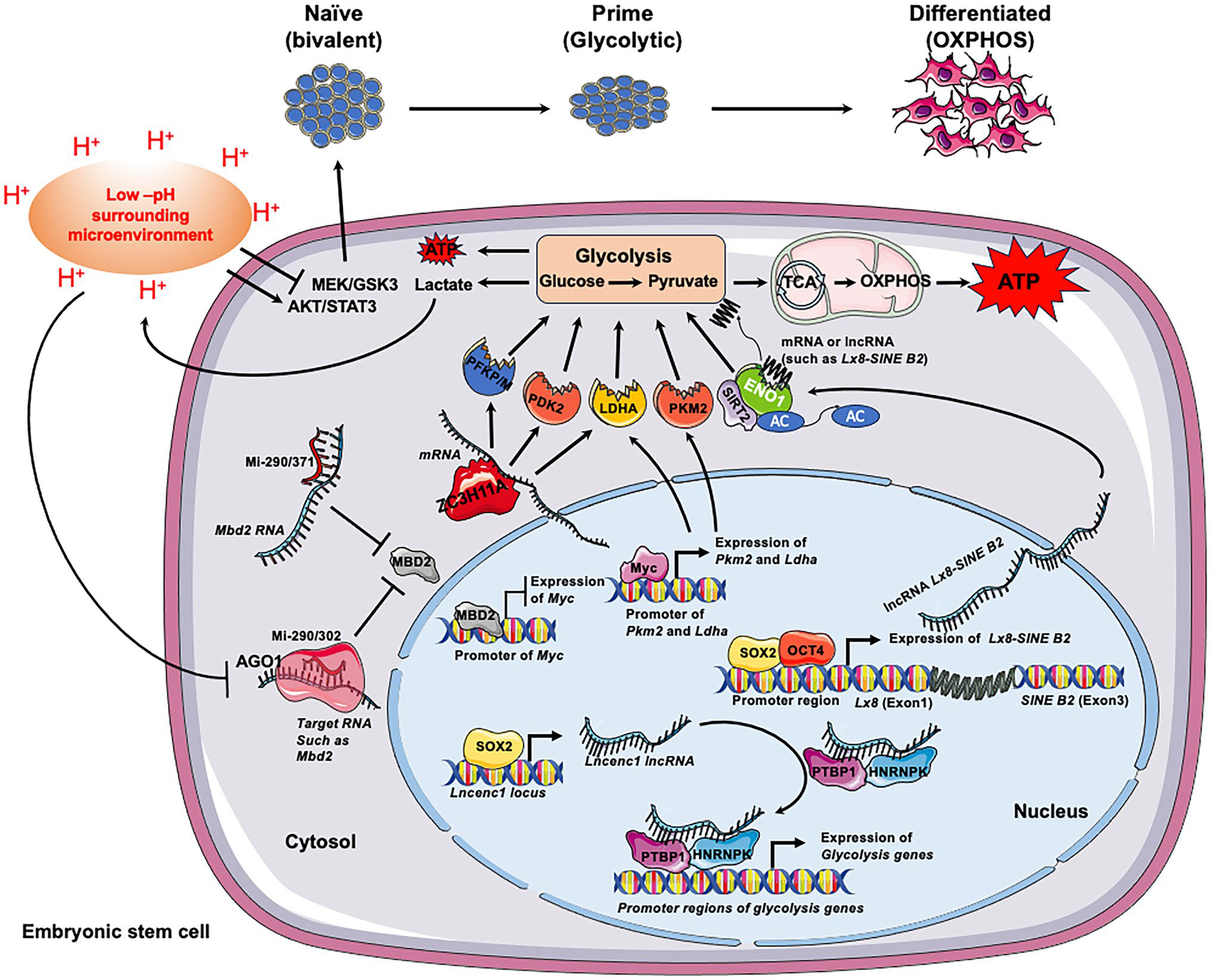
Riboregulation of glycolysis modulates self-renew, pluripotency and differentiation of ESCs. The pluripotency and differentiation states of ESCs are strictly coordinated with their metabolism statuses. Transition of ESCs from the naïve to the primed state, then to germ layer lineage is associated with metabolic shifts from bivalent metabolism, to exclusive glycolysis, and then to OXPHOS. Multiple types of RNA molecules and RNA binding proteins are involved in regulation of glycolysis in ESCs, which in turn impacts their maintenance and differentiation. First, many lncRNAs highly expressed in ESCs, such as *Lncenc1* and *Lx8*-*SINE B2*, are under direct transcriptional regulation of the core pluripotency regulators, including SOX2 and OCT4. They function to either promote the transcription of glycolytic enzymes by binding to RBPs or directly bind to and promote the activity glycolytic enzymes (e.g. ENO1), thereby are important for maintenance of pluripotent ESCs. Secondly, many glycolytic enzymes, particularly ENO1, are RBPs. Depending on specific RNA molecules, binding of RNAs could either inhibit or promote the activity ENOs, which in turn modulate glycolysis and pluripotency/differentiation of ESCs. Thirdly, miRNAs are essential for ESC proliferation and differentiation at multiple levels, including their transcription (via MBD2-c-MYC), posttranslational modification (via SIRT2), and environmental sensitivity (the acidic pH). Finally, RBPs (e.g. ZC3H11A) that mediate the nuclear export of mRNA of glycolytic enzymes are important for energy metabolism thereby maintenance of pluripotent ECSs

Sun et al. recently identified an RNA transcript in length of about 3400 nucleotides of *Lncenc1* gene as a key regulator of the naïve state of mESCs [22]. By using ribosomal RNA-depleted RNA sequencing (RNA-seq) analysis, Sun et al. found that *Lncenc1* lncRNA is highly abundant in mouse nESCs but is almost undetectable in EpiLCs. Deficiency of *Lncenc1* gene leads to reduction of *Oct4* and *Nanog* expression and deformation of clone morphology. Interestingly, *Lncenc1* deficiency also results in decreased expression of glycolysis genes and substantially impairs cellular glycolytic activity [22]. Conversely, overexpression of *Lncenc1* in nESCs increases the mRNA levels of glycolytic genes and delays their differentiation for one day after 2i/LIF removal. Mechanically, the core pluripotency factors, such as SOX2, directly regulate the expression of *Lncenc1* by binding to its locus. The lncRNA transcript of *Lncenc1* then forms a complex with RNA binding proteins polypyrimidine tract binding protein 1(PTBP1) and heterogeneous nuclear ribonucleoprotein K (HNRNPK) to control the transcription of glycolytic genes and subsequently the self-renewal of nESCs (Fig. 1) [22, 33].

Many lncRNAs derived from transposable element (TE), such as retrotransposon HERVH derived lncRNA, also act as important pluripotency regulators in ESCs [34, 35]. Chen et al. found that lncRNA *Lx8*-*SINE B2*, which has overlapping exon1 and 3 with neighboring transposon *LINE1 family Lx8* and *SINE B2* in genome and contains a long intergenic non-coding RNA (lincRNA) lincRNA-1282, is highly abundant in ESCs and a marker of pluripotent mESCs [35]. Its expression is downregulated during ESC differentiation or when cultured in serum containing medium, but is upregulated in 2i containing medium [35]. They further showed that core pluripotency regulators OCT4 and SOX2 directly bind to a TE ORR1D2 in promoter region of lncRNA *Lx8*-*SINE B2*, activating its expression in mESCs, which is functionally important for the expression of another key pluripotency factor c-MYC [35]. Through GO and KEGG analysis of RNA-Seq data, Chen et al. recently found that depletion of *Lx8*-*SINE B2* also results in downregulation of genes in multiple metabolic pathways, particularly glycolysis, leading to impaired glycolysis in mESCs [24].

## Riboregulation of Enolase 1 (ENO1) Modulates Glycolysis in ESCs

ENO1, a highly abundant glycolytic enzyme in mammalian cells, functions to catalyze the interconversion between phosphoenolpyruvate (PEP) and 2-phosphoglycerate (2-PG) in glycolysis [36–38]. Using T4 polynucleotide kinase (PNK) assay and enhanced crosslinking and immunoprecipitation (eCLIP), Huppertz et al. recently discovered that in highly glycolytic Hela cells, ENO1 interacts with a wide range of RNAs, including hundreds of mRNAs, through specific RNA binding domains [25••]. Interaction of ENO1 with these RNAs significantly interferes its enzymatic activity, as the synthetic RNA ligands dimmish the lactate production in Hela cells and alter metabolites in glycolysis and serine synthesis in mESCs [25••]. ENO1 is a known acetylated protein and an NAD^+^-dependent protein deacetylase SIRT2 has been previously shown to be the major deacetylase of ENO1 [39, 40]. In this study, Huppertz et al. discovered that the RNA binding capability of ENO1 is modulated by its acetylation status. In Hela cells, acetylated ENO1 maintains the RNA binding ability whereas SIRT2-mediated deacetylation represses this ability. Functionally, they showed that this RNA binding-mediated riboregulation of ENO1 affects differentiation of mESCs. The glycolysis to OXPHOS metabolic rewiring during mESC differentiation is associated with increased ENO1 acetylation, induced ENO1-RNA binding, and reduced ENO1 activity [25••]. Moreover, engineered mESCs expressing an ENO1 mutant with enhanced RNA-binding ability display significantly impaired germ layer differentiation, especially toward definitive endoderm and neuroectoderm [25••]. However, given that SIRT2 is known to be induced during differentiation of hESCs and suppressed during pluripotency reprogramming of human fibroblasts [39], this deacetylase is unlikely responsible to the increase in the acetylation levels of ENO1 during differentiation of mESCs observed in this study. Additional experiments are needed to identify the ENO1 acetylases and deacetylases involved in the regulation of mESC differentiation.

LncRNA *Lx8*-*SINE B2*-mediated metabolic regulation of pluripotency also involves riboregulation of ENO1 [24]. Unlike most RNAs transcribed from TE, a dominant portion of lncRNA *Lx8*-*SINE B2* is in the cytoplasm, where it binds to ENO1. The interaction with *Lx8*-*SINE B2* increases the expression of ENO1, hence promoting glycolysis and self-renewal of mESCs [24]. Therefore, depending on specific RNA molecules (e.g. mRNA vs lncRNA *Lx8*-*SINE B2*), riboregulation of ENO1 could either inhibit or promote its activity, which in turn modulate glycolysis and pluripotency/differentiation of ESCs.

## MicroRNAs in Regulation of Glycolysis and Pluripotency in ESCs

MicroRNA (miRNA), small non-coding RNA molecules generated from RNA polymerase II (Pol II) transcribed longer gene precursors, can post-transcriptionally silence the expression of multiple genes by inhibition of protein translation and/or promotion of mRNA cleavage [41, 42]. Using mouse models and mESCs lacking miRNA processing proteins such as DICER1 or DGCR8, previously studies have demonstrated that mature miRNAs are essential for ESC proliferation and differentiation [43–46]. Recently studies have shown that many miRNA molecules, such as members of the miR-200 and miR-290 family, are able to regulate the pluripotency by dictating metabolic preference of glycolysis over OXPHOS in PSCs [39, 47].

Cao et al. recently uncovered an essential function of the canonical miRNA molecules of miR-290 family (or its human homolog mi-371 family) in induction of multiple glycolytic enzymes, including pyruvate kinase 2 (PKM2) and lactate dehydrogenase A (LDHA), and thereby enhancing glycolysis in mESCs [47]. They showed that protein synthesis of MBD2, a transcriptional repressor, can be post-transcriptionally repressed by the miR-290/371 cluster. Since MBD2 binds to the promoter of *c*-*Myc* to inhibit its transcription in mESCs, miR-290/371-mediated repression of MBD2 enhances the expression of c-MYC, which in turn binds to the prompter regions of both *Pkm2 and Ldha* genes, activating their expression and boosting glycolysis (Fig. 1). This regulatory circuit enhances the glycolysis to promote maintenance of pluripotent ESCs and facilitates metabolic switch to promote reprogramming of human somatic cells to iPSCs [47].

Cha et al. recently showed that a member of the miR-200 family known to be induced by OCT4 [48], miR-200c-5p, is able to repress both mRNA and protein levels of SIRT2 in hPSCs [39]. As SIRT2-induced deacetylation of multiple glycolytic enzymes, including aldolase, GAPDH, ENO1, and PGK1, has been reported to inhibit their activity during the reprogramming of fibroblasts to iPSCs [39], miR-200c-5p may help to promote metabolic reprogramming during human induced pluripotency by repressing SIRT2.

microRNAs also play a role in mediating the impact of glycolysis on pluripotency in ESCs. In addition to generate anabolic intermediates for biosynthesis required for fast proliferation [14, 49], the highly activated glycolysis in ESCs also produces a large amount of lactate which is released and accumulated to extracellular space of ESCs. A recent study by Guo et al. showed that the released lactate from mESCs is sufficient to decrease of pH of extracellular environment in unbuffered media from 7.4 to 6.3 in 48 h [50•]. This reduction of pH can help to retain the expression of pluripotency markers, such as OCT4, NANOG and SOX2, and partially block the differentiation of mESCs and hESCs induced by medium without 2i/LIF and bFGF [50•]. In searching of the underlying molecular mechanisms, Guo et al. found that AGO1, a member of the argonaute protein family that interacts with miRNA to alter protein synthesis and affect RNA stability through RNA-based silencing mechanisms [51, 52], is significantly decreased by acidic-pH treatment [50•]. Furthermore, depletion of AGO1 increases expression of many pluripotency markers, including *Essrb*, *Klf2*, and *Nanog*, at an early timepoint of mESCs differentiation. Therefore, acidic pH-induced repression of AGO1 could partially explain its differentiation blocking function. Interestingly, they further found that the expression of many targets of miR-294/302, the most enriched miRNA in mESCs, is significantly increased by the acidic-pH treatment. One of these targets is *Mbd2*, a transcriptional repressor known to repress the expression of *Myc* [47] (Fig. 1). As a result, the expression of *Myc* is repressed by low pH [50•]. As MYC is critically involved in maintenance of naïve ESCs and its expression is reduced during the transition from naïve to primed pluripotent state [53], this observation suggests that acidic pH-induced repression of AGO1 may facilitate the exit of naive pluripotency state (Fig. 1). Collectively, miRNAs are key mediators of the interactions between glycolysis and pluripotency in ESCs.

## Other RBPs in Regulation of Differentiation and Glycolytic Metabolism of ESCs

Previous studies have uncovered many RBPs that modulate metabolic shifting in stem cells [2]. A recent study by Younis et al. showed that a stress-induced mRNA-binding protein, zinc finger CCCH domain–containing protein 11A (ZC3H11A), is important for maintenance of normal energy metabolism of ESCs. ZC3H11A is required for efficient growth of many nuclear-replicating viruses (e.g. HIV (HIV-1), influenza A, human adenovirus, and herpes simplex virus 1) in human cells due to its critical role on maintaining nuclear export of mRNAs under stress conditions [54]. Younis et al. found that *Zc3h11a* is very highly expressed at the early stage of embryonic development. Inactivation or homozygous deletion of this gene in mice result in lethality and embryonic degeneration [26•]. They further discovered that deletion of *Zc3h11a* leads to dysregulated fatty acid metabolism and glycolysis in E4.5 embryos, along with reduced expression of many genes involved in regulation of cellular energy metabolism, including *Ldha*, phosphofructokinase, platelet and muscle (*Pfkp and Pfkm*) and pyruvate dehydrogenase kinase, isoenzyme 2 (*Pdk2*). All these genes are directly involved in glycolysis and lactate production [26•, 55]. Consequently, deletion of *Zc3h11a* disrupts mitochondrial membrane potential and ZC3H11A deficient ESCs are differentiated into epiblast-like cells [26•] Mechanistically, they showed that ZC3H11A interacts with mRNA-export proteins and binding to the mRNA transcripts in ESCs, and is involved in export and posttranscriptional regulation of selected mRNA transcripts of glycolytic enzymes [26•].

## Concluding Remarks

Differentiation of ESCs from naïve to primed stated and further to germ layer specification, is coupled with metabolic shifts from bivalent state to glycolysis and further to OXPHOS. As a key element in these metabolic shifts, glycolysis and its modulation critically affect different stages of ESC development. This article summarizes the recent knowledges on how different types of RNA molecules and RBPs orchestrate with cell signaling circuities to regulate glycolysis at different stages of ESCs. Given the dual capabilities of ESCs/PSCs in pluripotent differentiation and unlimited self-renewal, better understanding of cellular riboregulatory mechanisms of their maintenance and transitions may lead to novel RNA-based therapeutic inventions in regenerative medicine.
